# Meeting the multidimensional self: fostering selfhood at the interface of Creative Arts Therapies and neuroscience

**DOI:** 10.3389/fpsyg.2024.1417035

**Published:** 2024-09-25

**Authors:** Sharon Vaisvaser

**Affiliations:** School of Society and the Arts, Ono Academic College, Kiryat Ono, Israel

**Keywords:** the self, nested layers, self disturbances, Creative Arts Therapies, embodiment, predictive processing, brain-to-brain synchrony, integration

## Abstract

Intriguing explorations at the intersection of the fields of neuroscience and psychology are driven by the quest to understand the neural underpinnings of “the self” and their psychotherapeutic implications. These translational efforts pertain to the unique Creative Arts Therapies (CATs) and the attributes and value of the self-related processes they offer. The self is considered as a multi-layered complex construct, comprising bodily and mental constituents, subjective–objective perspectives, spatial and temporal dimensions. Neuroscience research, mostly functional brain imaging, has proposed cogent models of the constitution, development and experience of the self, elucidating how the multiple dimensions of the self are supported by integrated hierarchical brain processes. The psychotherapeutic use of the art-forms, generating aesthetic experiences and creative processes, touch upon and connect the various layers of self-experience, nurturing the sense of self. The present conceptual analysis will describe and interweave the neural mechanisms and neural network configuration suggested to lie at the core of the ongoing self-experience, its deviations in psychopathology, and implications regarding the psychotherapeutic use of the arts. The well-established, parsimonious and neurobiologically plausible predictive processing account of brain-function will be discussed with regard to selfhood and consciousness. The epistemic affordance of the experiential CATs will further be portrayed, enabling and facilitating the creation of updated self-models of the body in the world. The neuropsychological impact of the relational therapeutic encounter will be delineated, acknowledging the intersubjective brain synchronization through communicative verbal and non-verbal means and aesthetic experiences. The recognition and assimilation of neuroscientific, phenomenological and clinical perspectives concerning the nested dimensionality of the self, ground the relational therapeutic process and the neuroplastic modulations that CATs have to offer on the premise of fostering, shaping and integrating selfhood.

## Introduction

Central to contemporary understanding of brain function is the role of the body, and its sensory-motor dynamic engagement and interaction with an information-rich environment, in the experience of the self (Gallagher et al., 2013; Fabiani, 2015; Kiverstein and Miller, 2015; Overgaard et al., 2023). This state-of-the-art neuroscientific perspective and its clinical applications point to the embodied, proactive and relational ways in which the brain operates, shoring up the reciprocal body-mind relationship (Vaisvaser, 2021). Indeed, recent developments in empirical and theoretical neuroscience highlight the experience-based interactive nature by which human beings, as biological entities, are embedded and shaped by the engagement with the environment and meaningful intra- and inter-personal contexts.

At the whole-brain scale, mental representations of the self, the other, and the world, dynamically evolve over time, through nerve impulses propagating in functional network assemblies (Pessoa, 2014). This is driven by the constant attempt to anticipate, decode and respond to a stream of sensory stimulations encompassing complex concrete and abstract variables, based on prior information and experiences (Ju and Bassett, 2020; Teufel and Fletcher, 2020). Neuroplasticity in that matter is well recognized as a fundamental and lifelong brain property, while plastic remodeling is an experience-based and activity-dependent phenomena, shown to rely on novel experiences, altered environmental input and the learning of new skills (May, 2011; Engel et al., 2016; Diniz and Crestani, 2023).

Using experience-oriented techniques, Creative Arts Therapies (CATs) carry out psychic work on fundamental aspects of the self and self-other interaction in diverse mental conditions and disorders, encompassing neuroplastic potential. CATs, such as dance/movement therapy, art therapy, music therapy, drama therapy, psychodrama, and bibliotherapy, are mental health professions that invite patients to proactively engage in their lived experience through multisensory creative processes, holistically integrating somatic, cognitive, emotional, cultural, aesthetic, and social aspects of the self (Dunphy et al., 2019). The art making process (whether encompassing visual, movement, music, drama or writing processes), consists of embodiment, creation, observation, reflection, and meaning making processes, aiming toward a better integration between body and mind (Lev-Wiesel and Kissos, 2019). Both verbal and non-verbal forms of expression and communication are used in CATs, accentuating embodied knowledge and memory, presence and liveness, and the transcendent qualities of imagination and creativity (Malchiodi, 2005; Van Lith, 2015; Czamanski-Cohen and Weihs, 2016; Gerber et al., 2018; Samaritter, 2018). The present manuscript aims at establishing a neuropsychological ground for implementation of such therapeutic work in the context of disturbances in selfhood and the crucial interplay of subjectivity and intersubjectivity.

## The predictive self-evidencing brain

The unifying well-founded Predictive Processing (PP) framework has emerged as a powerful and immensely influential paradigm for understanding how the neural system processes and dynamically generates models of the world and the self within it. This embodied and embedded framework of brain function highlights the importance of the interaction of agents with their environment, in perceptual, emotional, and cognitive domains (Allen and Friston, 2018). According to this framework, the brain continuously infers the hidden causes of internal (bodily) and external (physical and social) sensory stimuli, so that it can anticipate them and formulate an appropriate reaction, mostly on a subliminal or unconscious level. The framework builds upon the free energy principle, an extension of the idea that living organisms avoid the disseminating entropy, by engaging in self-organization (Schrödinger, 1956), aiming to maintain homeostasis to ensure survival and prosperity and minimize uncertainty (Friston, 2010; Clark, 2013; Hohwy, 2013).

The brain networks are hierarchically organized, from lower-level cortical sensory processing toward a functional spectrum of progressively higher-level associative and abstract representations (Wang, 2020). Correspondingly, in the context of PP, predictive signals descending (top-down) from higher-level processing areas are integrated with (bottom-up) ascending prediction error signals from sensory areas, refining these predictions and driving action on the surrounding environment to reduce its uncertainty (Parr and Friston, 2019).

Statistically, these processes entail approximate Bayesian inference, which combines sensory evidence (“likelihood”) and background knowledge (“prior probability”). The interaction and comparison between 'top-down' descending predictions, or expectations based on prior learning, and the ascending on-going somatosensorial information, lead to either a match or a mismatch in predictive models, i.e., prediction error (surprise). The minimization of free-energy, or cumulative precision-weighted predictive errors, may in turn lead to the updating of generative models (predictions) at higher levels. Accordingly, these salient prediction errors mediate attention (Feldman and Friston, 2010), and affordance—the latent possibilities for action (Picard and Friston, 2014).

This implies that what we perceive of the world and ourselves within it is subjective and geared to represent the environment in terms of embodied action opportunities as opposed to objective truth (Martin et al., 2021). Through active inference, embodied agents interpret and proactively interact with salient features of their environment (Bruineberg et al., 2018); which crucially includes other human agents (Bolis and Schilbach, 2020).

Along with multisensory perceptual inference in the exteroceptive domain, perceptual inference of the physiologic state of the body (interoception), or the moment-by-moment mapping of the body's internal milieu across conscious and unconscious levels, was suggested to underlie emotions and feelings, ensuring self-consciousness (Seth, 2013; Picard and Friston, 2014). Internal and external perceptual processes endure the predictive dynamics of increase and reduction of error (uncertainty), thus are inherently affectively charged. We thus live and prosper in the world we subjectively perceive and, moreover, we continuously perceive ourselves within it. Selfhood (or ipseity) is an agent's embodied model in the world, constantly searching for evidence for its own existence through its activities (Friston, 2010)—we are “self-evidencing” creatures (Hohwy, 2016).

The Conceptualization of the self as a self-organizing system implies that there is a need for an identifiable boundary condition. These boundaries are known as “Markov blankets”—the set of states that separate the internal parts of a system from its external ones (Cieri and Esposito, 2019). “Markov blankets” acts as a protective screen through which an organism is able to recognize and distinguish an internal side from an external environment, inferring the external or internal causes of sensations and perceptions. Selfhood (self-organizing existence within a world that can be separated from the self) is therefore dynamic and actively inferential. It involves the continuous model generating predictions about what is most likely to be “me”, as opposed to “not me” (Braun et al., 2018), through acting upon the world and sampling new sensory states, further conforming to goal or object-directed notions of affective intentionality (Solms and Friston, 2018).

## Dimensions of selfhood

The concept of the self has been explored from different perspectives in a variety of disciplines, and for diverse purposes, all of which converge on the dimensionality of the self, offering complementary insights. Extensive empirical and theoretical research aimed at elucidating the neural architecture related to selfhood and self-consciousness proposes nested dimensions attributed to different self-related processes. While some perspectives outline a hierarchical conception of the self, with the bottom layer providing the grounding for the constitution of higher levels, other views indicate the dynamic interactions between self-dimensions rather than considering a purely hierarchical organization (Raoul and Grosbras, 2023).

A basic distinction between two phenomenological aspects of the self, portrays an experiential and a narrated one (Zahavi, 2014; Davey and Harrison, 2022). While the first is the self that we experience in the present moment, the second is the self which becomes the object of our attention and reflection. The experiential aspect is ecologically and somatically embedded that forms our immediate subjective sense of being, its presence apprehended implicitly.

In accordance, seminal theoretical perspectives offer different views of the fundamental dimension of the self that resonate with the experiential essence, yet emphasize distinct affective, cognitive, bodily, or developmental experiences. These include the “proto-self” (Panksepp, 1998; Damasio, 1999, 2010), “minimal self” (Gallagher, 2000), “interoceptive self” (Qin et al., 2020), and the psychodynamic concept of an “autistic-contiguous position” (Ogden, 1989).

These pre-reflexive dimensions of embodied sense of selfhood relate to the sense of being a bodily subject or having bodily self-consciousness or self-awareness, which consists of ownership (the sense of the body as one's own, undergoing an experience), agency (the sense of the body as initiating and generating one's actions), first-person perspective (the feeling of seeing the world from one's own perspective), and self-location (the feeling that I am in my body) (Blanke, 2012). These aspects of the sense of self are thought to play an indispensable role in any self-experience (Gallagher, 2000).

Self-awareness is highly malleable and subject to the perception of the body from the *outside* as well as from *the inside* (Tsakiris, 2017). Therefore, it is influenced by sensorimotor and allostatic functions, or the monitoring and continuous anticipatory control of one's body state (homeostasis), which requires a model of the changing sensory conditions within the body, i.e., interoception (Sennesh et al., 2022). Indeed, the link between selfhood and interoception was suggested by Damasio and further demonstrated and established in extensive empirical studies (e.g., Aspell et al., 2013; Park et al., 2014; Park and Blanke, 2019). In concordance, the PP framework views emotion as “interoceptive inference”, related to embodiment as a basic feature of selfhood that is based on interception, proprioception and multisensory integration (Seth, 2013). Notably, interoceptive sensing of one's own body and exteroceptive sensing of the world are highly interdependent (Zaidel and Salomon, 2023). It is the predictive processing of crossmodal interactions and integration of multisensory signals—exteroceptive (Blanke et al., 2015; Salomon, 2017), volitional (Stern et al., 2020) and interoceptive (Seth et al., 2012; Park et al., 2016; Allen and Tsakiris, 2019), that was suggested to underlie the formation of coherent bodily self models (Clark, 2013; Limanowski and Blankenburg, 2013; Apps and Tsakiris, 2014; Zaidel and Salomon, 2023).

Accordingly, the self evolves due to the transient relationship between an individual and the surrounding environment, represented by Damasio's (2010) “core self”, and from an ecological phenomenological viewpoint referred to as “extero-proprioceptive Self” that actively extends to the outer body (Qin et al., 2020; Northoff and Scalabrini, 2021). This dimension of the self allows the collocation in space and time, and a more coherent self concept, dependent upon cross-modal multisensory integration.

Another important concept that should be considered in this regard is the space immediately surrounding the body, termed the peripersonal space (PPS, within reach), which is of foremost importance as it is the space in which physical interactions with the environment take place. The PPS represents a multisensory and sensorimotor interface mediating the physical interactions between the body and the environment (Pellegrino and Làdavas, 2015; Serino, 2019), encompassing emotional salience and social cognitive aspects (Basile et al., 2024). Accordingly, multisensory integration has been shown necessary to create and to maintain both an intact PPS and self-consciousness (Blanke et al., 2015). Studies have linked the actions in the PPS to a vast spectrum of functions including body representation, goal-directed movements, defensive movements, and social interactions (Geers and Coello, 2023). Pre-reflective selfhood thus emerges from one's experience through his/her body (Limanowski and Blankenburg, 2013; Apps and Tsakiris, 2014), with a clear link between self-related objects and a rewarding/hedonic experience, engaging the mesolimbic reward circuitry (De Greck et al., 2007).

Recent neuroscientific studies of these pre-reflective proto and core self-dimensions (mainly using virtual reality and clinical observations) suggest that the immediate self-consciousness of being the subject of experience, relies on information processed in the salience network, anchored in the anterior insula and anterior cingulate cortex, the somatosensory and sensorimotor cortices, inferior frontal gyrus (IFG), and temporo-parietal junction (TPJ) (Schaller et al., 2021; Crucianelli et al., 2024; Scalabrini et al., 2024); along with subcortical regions such as the amygdala, putamen and cerebellum (Crucianelli et al., 2024).

The sense of self-continuity across time, having a matrix of associations derived from experience in the form of autobiographical memories, further matures into the extended representational dimension of the self that was coined “autobiographical” (Damasio, 2010), “narrative” (Gallagher, 2000) or “mental” self (Qin et al., 2020). This is the dimension of the self that records the history of the individual, compares it to present experience, and prepares for the future. Self-referential processing and the temporal integration of self-experiences enable a sense of self identity (Scalabrini et al., 2022; Di Plinio et al., 2024). This forms the narrated aspect of self, which becomes the object of one's attention (Davey and Harrison, 2022), with cognitive symbolic abstraction abilities that allows experiences to be manipulated in imagination. Such self-experience was shown to be mediated by the Default Mode Network (DMN) that sits atop the hierarchy of neural processes (Molnar-Szakacs and Uddin, 2013; Qin et al., 2020). The DMN, which includes cortical midline structures- ventromedial prefrontal cortex (vmPFC) and posterior cingulate cortex (PCC) and recollection regions (hippocampus and precuneus), is involved in processes such as self-reflection, autobiographical memory, future event simulation, conceptual processing, mind-wandering, and spontaneous cognition (see Raichle, 2015 for a review). The vmPFC part of the DMN was mainly related to abstract self-representations (Hu et al., 2016). The DMN was suggested to integrate and broadcast memory representations to create a coherent “internal narrative” reflecting the individual's experiences, which is central to the construction of an extended sense of self, and the shaping of social interaction (Menon, 2023). The following section will delineate the meaning and means of integration between these dimensions of self-experience and their disturbance in psychopathology.

## Integration of self-dimensions in the brain-body-mind and its disturbance

The dimensions of the self are connected at their root and constructed upon one another, or dynamically interact with one another, as indicated in brain functionality (Raoul and Grosbras, 2023). Accordingly, brain regions associated with primitive dimensions of the self may be also related to the narrated dimension, where they are complemented by additional regions extending the topography of the self and its nestedness (Scalabrini et al., 2024). The anterior insula has been suggested to represent the common denominator of the nested self (Scalabrini et al., 2021), serving as a dynamic node or “the glue” between the different layers (Northoff, 2023). As part of the salience network, it subserves key functions of affective processing, monitoring interoceptive signals and connecting them with exteroceptive cues to create an integrative interface that enables embodiment, emotional mirroring, higher-order representation (Craig, 2009; Seth, 2013; Park et al., 2018), as well as the guidance of attention between internal and external directed cognition (Uddin, 2015; Harduf et al., 2023). Simultaneously, some DMN hubs track bodily oscillations, such as heartbeats, driving the cognitive level of self-related thought and agency of the experience (Babo-Rebelo et al., 2016). The functional connectivity between DMN and salience network supports a wide range of affective and cognitive psychological functions (emotion, memory, decision-making, etc.) that can all be explained by their reliance on interoception and allostasis (Kleckner et al., 2017).

Self-related spontaneous thought is known to be important for health and psychological well-being, it arises from episodic and affective processes, and its regulation relies on executive control (Smallwood and Schooler, 2015). The salience network that plays a key role in identifying relevant stimuli from the vast and continuous sensory stream, was proposed to switch between the DMN and the Executive (or frontoparietal) Control Network (ECN), facilitating the dynamic flexible transition from self-referential mental processes to the current task goals. A mechanism that is disrupted in psychiatric and neurological disorders (Menon, 2011; Menon et al., 2023).

Accordingly, alterations in vmPFC and interconnected areas of the DMN have been implicated in multiple psychiatric conditions, including major depressive disorder, anxiety, schizophrenia, attention deficit–hyperactivity disorder, post-traumatic stress disorder and substance-use disorders (Hiser and Koenigs, 2018). The vmPFC was also shown to be functionally connected to core regions of the reward network (like the ventral striatum), involved in basic motivational processes during self-referential processing (D'Argembeau, 2013). Indeed, the multidimensional sense of self is rooted in brain network interactions. A coherent sense of self emerges from and relies on functional integration and segregation of default mode, sensorimotor, salience and executive brain networks (Di Plinio et al., 2020), disturbed or abnormal in disorders of the self.

Temporality, the subjective experience of time, pertains not simply to experiences of time passing but to a broader variety of temporal features inherent to the lived experience, for instance, the unfolding of the experience as a stream of consciousness, as opposed to being discontinuous and fragmented (Kyzar and Denfield, 2023). The temporal continuity of self can be defined as how we perceive and experience ourselves (including the body and mental states) as one and the same across time in mental time travel (Beaty et al., 2019). Neuroscience indicated similarity of the neurological underpinnings of past- and future-oriented cognitive processes, attributed mainly to the DMN function (Karapanagiotidis et al., 2017). Neuropsychological evidence connects the disturbances of temporal integration and DMN function with psychopathologies, such as the slowness of time in depression and time fragmentation in schizophrenia (Northoff and Hirjak, 2022). The temporal-dynamic and spatial-topographic confusion of internal and external activity/events in schizophrenia may be shared by both experiential and neural levels (Northoff et al., 2023). The salience network was also shown to be pivotal in time experience, from its central role in interoception, to high-order processes, such as knowledge about oneself and the self extended in time, namely autobiographical memory (Tisserand et al., 2023).

The fragility of the self is emphasized by the “self-evidencing” perspective, based on PP, viewing the self as being “the most parsimonious and accurate explanation for sensory inputs” (Fotopoulou and Tsakiris, 2017, p. 10). The experience of self-coherence and self-continuity in this account is interestingly considered as an “illusion” that is the product of the capacity for mentalizing, created “on-line” and in contrast with what is “not me” (Luyten et al., 2021). Impairment in embodied mentalizing is associated with a rigid focus on either “self” or “other,” with detrimental effects on capacities for emotion co-regulation, social learning, and self-stability (Luyten et al., 2021).

The primordial pre-reflective self-constitution is disrupted in “primitive mental states” that may arise in psychosis, autism, borderline personality and in traumatic circumstances, in which patients present fragility in implicit first-person perspective and presence, aberrant sense of the self as an experiencing subject, with altered sense of agency or ownership of one's actions, perceptions and thoughts, as well as instabilities in self-other boundaries and differentiation (Nelson et al., 2014, 2020; Parnas and Zandersen, 2018; Feyaerts et al., 2021; Park and Baxter, 2022). Another related phenomenon of minimal self disturbances is the “split” of the self, with disrupted integration between signals from within the body (interoceptive) and signals from the external environment (exteroceptive), as seen in depersonalization (Saini et al., 2022).

Individuals with schizophrenia spectrum disorders show source monitoring deficits, which refer to difficulties in making attributions about the origins of self-experience, whether real or imagined, or whether originated internally or externally, critically relevant for diminished sense of ownership and agency (Feyaerts and Sass, 2024). Disturbed functional connectivity and corollary discharge make actions or thoughts unpredictable and, consequently, not attenuated as self-generated sensations normally are (e.g., tickling oneself) (Blakemore et al., 2000; Abram et al., 2022). This leads to difficulties in distinguishing events that one may control from events that occur in the external world. The insula-anchored salience network was suggested to play a role in the misattribution of aberrant salience to irrelevant external stimuli and self-referential mental events, leading to connections being made between unrelated events and possibly, over time, delusional thinking (Menon et al., 2023). Consequently, internal thoughts and actions may be misattributed to external agents, evolving into psychotic positive symptoms such as hallucinations and passivity phenomena (the feeling that an external agent is controlling one's body, feelings or thoughts) (Gaweda et al., 2013). Aberrant activity and connectivity within and between the salience network, DMN and ECN (reviewed by Menon et al., 2023), and associated disturbances of memory, prediction and attention processes, were suggested to contribute to hyper-reflexivity, disturbed “grip” or “hold” on the perceptual and conceptual field, and disturbances of intuitive social understanding (Sass and Feyaerts, 2024).

In autism spectrum disorder, repetitive motor behaviors and insistence on sameness (i.e., behavioral rigidity and resistance to change), were related to anxiety and intolerance of uncertainty (Wigham et al., 2015). These restrictive and repetitive behaviors were explained by atypical sensory processing, lack of multisensory integration and reduced connectivity within and between networks related to motor function, saliency and attention (Llioska et al., 2023). Repetitive behaviors provide a way to manage atypically regulated sensory stimulation by means of reducing overstimulation or creating stimulation in cases of stimulation seeking (Wigham et al., 2015). This may also explain self-stimulation (stimming) behaviors, when individuals perform a repetitive and highly predictable “self-evidencing” action upon themselves (Perrykkad and Hohwy, 2020).

Disordered self-processing coincides with disturbances in PPS representation that have been increasingly documented in various neuropsychiatric disorders, including schizophrenia and autism, and linked to social impairments. Typically, dynamic and flexible boundaries exist between the PPS and the extrapersonal space, i.e., the area further from the body, where objects cannot be reached, and the (social) environment is explored. Interestingly, smaller PPS with sharper (steeper) boundaries was found in the autistic spectrum, suggested to point toward a rigid self-other differentiation, while blurred and shallow PPS boundaries in the schizophrenia spectrum reflects a weaker self-other distinction (Noel et al., 2017).

Furthermore, the decreased behavioral flexibility and difficulties in navigating dynamic real-world social situations in autism, was associated with aberrant connectivity within the DMN and between the DMN and the rest of the brain, particularly with the ECN (Llioska et al., 2023). The abnormal DMN function may be viewed as a lack of access to the upper dimension of the 'mental self', related to the weak self-referentiality and self–other distinction (Lian and Northoff, 2021). On a cognitive level, atypical network integration and segregation may result in abnormally increased rationality and systematizing in cognition, i.e., hypersystematizing (Baron-Cohen and Lombardo, 2017). This points to the importance of acknowledging neurodiversity (Baron-Cohen, 2017), a perspective that considers variations in brain structure and function, leading to a different experience of the self and the world. The neurodiversity movement advocates for interventions delivered in a person-centered manner (Den Houting, 2019), corresponding with CATs therapeutic orientation.

Importantly, autism and schizophrenia spectrum disorders need to be recognized in their diversity, due to the heterogeneity of symptoms and neural correlates (Sass and Feyaerts, 2024). Individual differences also relate to the concept of neurodiversity, pointing to the importance of condescending to the patient's perspective by viewing it not only as inaccurate but as somehow different than that of the non-psychotic or neurotypical individual, considering the contextual, historical and relational background within which meaning is co-constituted (Ritunnano, 2022). This conceptualization aligns well with the therapeutic emphasis in CATs on the bodily intra- and inter-personal “lived experience”, further developed in the sections below.

Trauma exposure may induce dysfunctional generative models, with long-term effects as seen in Post Traumatic Stress Disorder (PTSD), including hyperprecise aberrant predictions of threat that trigger defensive responses, as well the disconnection of the self model from the model of the traumatic event in dissociative symptoms, manifesting in the failure to update models based on ascending interoceptive predictive errors (Wilkinson et al., 2017; Linson and Friston, 2019; Krupnik, 2020).

Certain types of trauma may have differential impact on brain patterns and clinical manifestations. It has been recently shown that individuals with relational attachment-based traumatic experiences mainly recruit regions belonging to lower layers of the self, mainly the salience network, suggested to indicate that their sense of self doesn't reach a higher symbolic capacity, interpreted as a more primitive form of affective regulation (Scalabrini et al., 2024). Brain network organization of maltreated individuals may result in a diminished capacity to regulate impulses and emotions, accurately attribute thoughts and intentions to others, and to be mindful of oneself in a social context (Teicher and Samson, 2016).

Individuals with trauma-related dissociative disorder were shown to implement neural mechanisms of cognitive control (hyperactivation of ECN), along with hypoactivation of salience regions, suggested to serve as protective means to mentally avoid self-relevant (interoceptive) processing of trauma-related knowledge (Dimitrova et al., 2024). Torture survivors showed increased ECN-DMN dynamic functional connectivity suggesting top-down cognitive control over internal self-referential emotional processing, suggested as an adaptive over-regulation response, with possible consequences including emotional rigidity, inflexibility, and withdrawal (Liddell et al., 2022).

Individuals diagnosed with PTSD and concurrent moral injury (i.e., the experience of causing, witnessing, or failing to prevent an action that contradicts an internalized, embodied sense of morality), show hyperconnectivity of the default and sensorimotor networks (Kearney et al., 2023). The diminished network segregation and enhanced recruitment of sensorimotor regions during traumatic retrieval offer a neural basis for putative sensorimotor imprints which lack appropriate integration into autobiographical narratives, thus contributing toward distressing symptoms of re-experiencing or re-enactment (Kearney et al., 2023).

Notably, the effects of trauma on neuropsychological functioning and the clinical profile are likely to differ depending on various factors such as trauma dosage, age of the traumatic experience, type of trauma, and the chronicity of the trauma (Teicher and Samson, 2016). As aforementioned, heterogeneity and individual differences need to be accounted for and considered in the formulation and treatment of disturbances or disorders of the self. The development of novel psychometric tools capable of measuring individual differences in fundamental aspects of self-experience may be of great value in the study of mechanisms and treatment of various disturbances of the self (Di Plinio et al., 2024). The CATs are both personalized and process-oriented, as are the therapeutic relationship and transference/countertransference dynamics, thus considering subjective individual differences.

The following sections will describe the interpersonal foundation of the self and ways in which the psychotherapeutic relationship can foster, support and enhance self-related processes.

## Relational selves sync

The regulation of interoception and allostatic needs that lie at the core of the self, is initially and continuously supported by the sensitively attuned attachment figure (Atzil et al., 2018). Predictable input from the caregiver enables the brain to begin differentiating “self” vs. “non-self” causes of sensations, securing an experience of ownership and agency (Fonagy et al., 2002; Seth, 2013; Di Plinio et al., 2020). The embodied interaction allows the “mentalization” of visceral sensation as experienced subjective feelings, further establishing mental self-processing. These are processes of interpersonal match/mismatch and repair, also named “mentalizing homeostasis” (Fotopoulou and Tsakiris, 2017). The embodied “dialectical attunement” ground the higher-order abstract thinking, considered a re-enactment of the internalized dialogue through which one acquired the concepts in the first place (Bolis and Schilbach, 2020; Fini et al., 2023). The embodied attunement applies to the psychotherapeutic relationship, forming emotional communicating and regulating bond between the patient and the empathic clinician (Schore, 2022).

Shared interactive moments extend beyond the physical boundaries of one's individual brain and induce brain-to-brain interpersonal neural synchrony, identified through multi-brain recording approaches, i.e., the use simultaneous recordings from two or more interacting individuals (i.e., hyperscanning) in two-person/second-person neuroscience (Hari et al., 2015; Redcay and Schilbach, 2019). Synchronized brain function was suggested as a mechanism of transmission of shared meaning, intentionality and interpretation in dyads and in groups, whereas aesthetic experiences were repeatedly shown to induce intersubject synchrony (e.g., Müller et al., 2013; Reddish et al., 2013; Sachs et al., 2020; da Cruz Monteiro et al., 2022; Greaves et al., 2022; Tucek et al., 2022), also recently shown CATs (Samadani et al., 2021; Tucek et al., 2022; Kang et al., 2023). Individuals also become physiologically attuned as their autonomic nervous systems synchronize as measured by heart rate, breathing patterns, skin conductance, and hormonal endocrine responses (Palumbo et al., 2016). Advances in mobile brain/body imaging technology may provide extensive neurophysiological understandings in natural settings and in therapeutic relational contexts, enabling freely moving behaviors (King and Parada, 2021; Stangl et al., 2023; Vaisvaser et al., 2024).

In this regard, the CATs integrally incorporate behavioral entrainment, or rhythmic synchronization between the attuned therapist and the patient (Malchiodi, 2023). This may enhance reciprocity and mutual engagement, forwarding integration as well as relational processes and communication (Trevarthen and Aitken, 2001). Indeed, biobehavioral moments of synchrony are critical in the psychotherapeutic setting, fundamental in the dialectical process between self and other which shapes the sense of self (Scalabrini et al., 2022). Spontaneous interpersonal neural synchrony may emerge from behavioral synchrony during social interaction (Koul et al., 2023), fostered by the relational aesthetic engagement in CATs (Vaisvaser et al., 2024).

Interpersonal interaction and communication depend on shared predictive models (Rosso et al., 2022; Gallotti et al., 2017). Accordingly, brain-to-brain synchronization was suggested to entrain shared PP, whereby the oscillatory synchrony is viewed as an emergent property of free energy minimization (Friston, 2010; Friston and Frith, 2015). This implies that within meaningful interpersonal engagement, we not only predict each other, but we (en)actively entrain our perceptual and motor states. Individuals that are coupled via sensory information and interact on a physical, emotional and mental level were suggested to form a shared entity of active inference and precision-weighting, such that the ontology of their brain function becomes one (Friston and Frith, 2015). The emergence of this unifying “narrative” (generative predictive model) may change each person's perception. Accordingly, psychopathology can be viewed not as mere (mis-)function within the single brain of the self, but also as a dynamic interpersonal mismatch (Bolis et al., 2023). Indeed, many forms of psychopathology are associated with a reduced ability to achieve brain-to-brain synchrony, and it has been suggested that recurring exposure to high inter-brain synchrony in therapy may lead to plastic changes and lasting changes in a person's overall ability to synchronize (Sened et al., 2022). These moments of intersubjective neuropsychological synchronization promote development and resilience (Feldman, 2020). The next section deepens into the psychotherapeutic processes in CATs that hold promise for cultivating and integrating the different dimensions of the self.

## Nurturing the self in Creative Arts Therapies

Psychotherapy aims to touch upon, explore, cultivate, and integrate the different aspects of the self. The embodied, experiential, exploratory, active, enactive, and integrative nature of CATS may offer novel psychotherapeutic avenues for a broad range of disturbances of the self. Aesthetic experiences are a concomitant part of CATs, in which the creative process that encompasses the interplay between expression and impression, promotes therapeutic factors of externalization-concretization, embodiment, and symbolization, facilitating affective and cognitive processing (de Witte et al., 2021; Vaisvaser et al., 2024). Aesthetic neurodynamics resonate with an individual's sense of self, involving the crosstalk between the DMN and both sensorimotor cortices and the salience and reward networks, as well as the dialectical interactions between the DMN and ECN (Belfi et al., 2019; Chatterjee and Vartanian, 2016; Vessel et al., 2013; Vaisvaser et al., 2024). The CATs provide a safe environment for sensory exploration, promoting the development of interoceptive self-awareness, emotional embodiment, and cognitive understanding through the processing of (working through) the aesthetic experience (Samaritter, 2018; Vaisvaser, 2021; Vaisvaser et al., 2024). The individual's engagement with the arts generates dynamics that drive the inference of the hidden causes in the subject's internal and external environment, aligning with the action-oriented PP framework of brain function (Vaisvaser et al., 2024).

According to the PP framework, agents aim to reduce the discrepancy between expectations and observations, to maximize intrinsic or epistemic value, and (enactively) choose the actions that hold promise to reduce uncertainty about the states of the body in the world. Beyond homeostatic adaptation to the ever-changing environment, humans are also curious and seek surprise and novelty in non-threatening settings (Clark, 2018). Indeed, PP renders novelty seeking and responding to epistemic affordance, or possibilities for action the environment offers, a natural part of the way we forage for information in the service of self-modeling (and self-evidencing) (Picard and Friston, 2014; Köster et al., 2020). Importantly, such information foraging is greatly facilitated by aesthetic objects (e.g., an artwork, music, the body) and experiences (Van de Cruys, 2017), during which fluctuations in the (un)certainty of predictions create an affective “entropic flux” (Koelsch, 2014). Such seeking behaviors in creative and aesthetic experiences recruit the mesolimbic dopaminergic reward system (Kenett et al., 2023). Immersion in aesthetic experiences, such as when engaging in CATs, may provoke altered states of consciousness (Vroegh, 2024), allowing for the experiential self to thrive (van Mulukom, 2021). These processes may optimize “free energy”, reduce the precision of rigid self-related prediction priors, and enable access to a range of alternate hypotheses that underwrite how one experiences and makes sense of the world. The psychotherapeutic use of the arts involves critical transitions between immersion (first-person perspective) and distancing (third-person perspective), allowing for a more flexible, adaptive, integrated self experience (Barbosa et al., 2021).

CATs may thus tap into the intrinsic motivation to seek predictive progress and offer patients opportunities to venture out of their predictable zones (Gottlieb et al., 2013; Vaisvaser, 2021). The aesthetic experiences in CATs capture the essential tension between stability and change and cultivate afford refined possibilities for self-restructuring and structuring of the sensed world (Van de Cruys et al., 2023). In a stable setting, they draw attention and drive exploration further, through an epistemic promise that one will be able to resolve uncertainty, while working on his/her percepts and letting them work on him/her, promoting sense making (Van de Cruys et al., 2023). Learning through these intrinsically motivating and curiosity-driven activities, progresses to yield an improvement of prediction and thus a reduction in uncertainty in the long-term (Friston et al., 2012; Kidd and Neuron, 2015). Such activities may be termed “progress niches” (Oudeyer et al., 2007), in which the progress in learning itself generates intrinsic rewards and an action selection system directly aimed to maximize this reward. These experiences soliciting epistemic affordances will determine the direction of future anticipation of affordances (van Dijk and Rietveld, 2021). Affordances in aesthetic encounters may emerge as opportunities to carry out an active exploration both at the pre-reflective and the reflective side of experience, as transformative events. These may be accounted as “opportunities to modulate, sooth, enhance, rewrite, explore, feel, forget or merely reflect upon aspects of the narrative self, such as memories, interests, likings, desires or habits in a socially situated, extended, intersubjective, and embodied context” (Vara Sánchez, 2022, p. 329). Such processes, involving the DMN, have profound impact on self-awareness and may significantly change people's relationship with the social context of which they are a part (Vara Sánchez, 2022). Notably, epistemic affordances rely on epistemic trust (Fonagy and Allison, 2014). Using their own mentalizing capacity therapists provide a secure relational base facilitating attachment and exploration. Within the context of a secure therapeutic environment, through artistic communication and creativity, the resolution of uncertainty is explored through epistemic foraging, novelty and shared emotional experiences (Schmidhuber, 2012; Friston et al., 2017).

In CATs we aim to enlarge and enrich the action space, or the space of affordances, facilitating intrinsic motivation, Bayesian surprise, or salience. The patient is offered the opportunity to organize a set of internal representations (predictive models) through an externalized aesthetic object supporting the projection of internal associativity. The therapeutic session forms a containing space for an increase in “free energy”, allowing the subject to safely make prediction errors and confront surprise effects. Through their echoing and their own capacity for uncertainty or “negative capability” (Bion, 1965) therapists will favor the effects of surprise, uncertainty and play in patients. This is what allows the reduction of the precisions of the patients' prediction priors, making it possible to “open up” to experiences, emotions, and thoughts that they did not have before (Connolly, 2022).

The therapeutic process in CATs induces perceptive awareness, including both interoceptive and exteroceptive awareness (Abbing et al., 2023). The psychotherapeutic use of multisensory processes is of great importance when considering the multisensory nature of self representations in the brain (Tsakiris, 2017). Multisensory integration of interoceptive, proprioceptive, and exteroceptive inputs, attributed mainly to the insular cortex activity and functional connections, supports body-ownership and agency over one's actions (Blanke et al., 2015; Salvato et al., 2019). In accordance, the insula has consistently been found to be active across aesthetic experiences in different sensory modalities (Brown et al., 2011).

Importantly, the interpersonal relational context in which aesthetic experiences emerge within the CATs, may engender intersubjective synchronization (Vaisvaser et al., 2024). Clinically relevant shared emotional processing reflected in interpersonal synchrony, was suggested to emerge from the coupling of active inference systems, underscoring the importance of embodiment and dynamical interaction (Gallagher and Allen, 2018).

Indeed, the use of one's body in the interactive constellation of CATs encompasses pivotal value. Experiencing oneself as undergoing an experience and being the source of her/his own motor actions is central to the phenomenal experience constituting self-consciousness, facilitating ownership and agency (Gallagher, 2000; Gallese and Sinigaglia, 2010). The aesthetic engagement may induce plasticity of body and PPS representations, especially in an interpersonal context (Drummond, 2024). The relational context endorses a shared process of predictive multisensory integration of events occurring in one's own action and may support predictions of the other's behavior and mutual adjustments during motor interactions (Fanghella et al., 2021). Moreover, self narratives arise from the lived experience, anchored in embodied activity. In traumatic circumstances and primitive mental states, CATs may enable to create narratives generated by the body and the art materials, these may be pre-symbolic, implicit and relational unconscious or unrepresented materials that have never been narrated by conventional means (Gallagher and Hutto, 2018).

The CATs enable the processing of the spatial and temporal aspects of selfhood. The body we work with (simultaneously) represents one's past, present, and future (concretely and symbolically), and creations are associated with expressions of time perception (Lev-Wiesel and Kissos, 2019). Moreover, aesthetic experiences have been shown to induce a sense of continuity or connectedness among past, present, and future selves (Pan and Jiang, 2023).

CATs may thus be applicable and effective in all levels of mental functioning and disorders of the self. Primary self-disturbances may require an intensified “vitalizing” intervention (Alvarez, 2012), using body movement, art materials, sound or music to enliven interpersonal exchange and “reclaim” patients, engaging them in the world of emotions and relationships (Vaisvaser, 2019). Higher level mental self-related processes require symbolization. This symbolic transformation concerns the various stages and processes that allow psychic reality to transform a “primary psychic matter” (“free energy”) into a matter endowed with reflexivity and subjectivity (Rabeyron, 2022). The use of imagery, symbolism, and metaphor via verbal and non-verbal expressive forms in CATs enable patients to externalize and further process emotions and experiences that they may not have been able to express in plain words, facilitating meaning-making processes (de Witte et al., 2021). These creative processes nurture the formation of increasingly complex, embodied and coherent internal (generative) representations, or models of the self in the world.

CATs invite and address the dialectic tension between the concrete and the symbolic, the somatic and the mental. They fluctuate between an inward and outward focus of attention, the “here and now” as well as mental time travel, memory recollections and mind-wandering; cultivating the capacity to engage in both poles (verbally and non-verbally) with more flexibility. These critical transitions and interactions lie at the core of psychological development and mental health. The processing of traumatic autobiographical memories may have a profound influence on individuals' wellbeing, as they have been suggested to be a transdiagnostic feature of multiple mental health difficulties (Dalgleish and Hitchcock, 2023). The multi-layered focus of therapeutic work and engagement in aesthetic experiences may soften the rigidity of model updating mechanisms, enabling autobiographical memory reconsolidation in light of corrective emotional relational experiences (Ecker et al., 2024; Vaisvaser et al., 2024).

## Summary and conclusions

The paper interweaves contemporary perspectives on brain function related to the self, providing an infrastructure for the translational effort to bridge neuroscience and subjectivity, further grounding the psychotherapeutic use of the arts. Main comprehensions of the nested dimensions of self-dimensionality, the PP framework and brain network functioning are interlaced to discuss their clinical meaning and applicability. The interdisciplinary integration allows us to deduce that the self-experience and mental representations are bound to our embodied existence in the world and that the affective actuality and lived experience are crucial in the therapeutic field. This scaffolds the neuropsychological plasticity associated with the formulation of CATs, promoting relational artistic and creative engagement that fosters the multidimensional sense of self.

The psychotherapeutic work entails active participation in motor, multisensorial, affective, cognitive and social aspects of bodily experiences, contributing to the experience of the self as a situated agent, extended over time and space. CATs may enable the working through process in primitive non-verbal and pre-reflective states and may invite creative processes of narration, symbolic formation and metaphoric work, promoting figurability (Botella and Botella, 2005). These processes may facilitate the functional crosstalk and integration of brain networks, prompting experience-dependent plasticity in neural circuitry that may provide opportunities to enhance resilience (Sened et al., 2022). Moreover, the creative experiences of the therapists themselves prompts the willingness and openness to the “zone of uncertainty” and surprise, deepening receptivity while inviting patients to step into unconscious raw experience, coinciding with Bion's “O” (“Absolute Truth”, or “Ultimate Reality”, Bion, 1965). While transformations in K (Knowledge) occur through processes of “talking about” or “understanding”, transformations in O (Origin), occur by processes of being (Sandler, 2018). Moving and being moved, CATs enable intersubjective 'now moments' of surprise and implicit relational learning (Boston Change Process Study Group, 2018), fundamental for play and creativity (Winnicott, 1971). As therapists, we are committed to the ongoing processes of self and self-other explorations, to transference-countertransference (including somatic) transactions, and interpersonal attunement driving inter-brain synchronization and plasticity within therapeutic relationships. Neuroscience-informed self and self-other referential processing may help deepen receptivity to the experiences being communicated, leading to new understandings with transformative potential.

The multi-layered processes that CATs offer enable the individualized formulation of each case that integrally takes into account the subjective experience of the patient, the dimensions of the self and the possibilities for interconnectedness. At the intersection of the fields of neuroscience and CATs, the psychotherapeutic use of the arts holds great promise to provide a receptive meeting ground for the multidimensional self, nurturing its processing and integration.
